# *Letter to the Editor:* The Need for Equitable Health Care Among Medical Cannabis Patients in Canada

**DOI:** 10.1089/can.2022.0084

**Published:** 2022-10-12

**Authors:** Meenu Minhas, Stephanie E. Lunn

**Affiliations:** Department of Medical Affairs, Aurora Cannabis Inc., Edmonton, Alberta, Canada.

**Dear Editor:**

As of February 2021, almost 300,000 Canadians are using medical cannabis for the management of various health ailments.^1^ The majority of these patients are paying out of pocket due to the lack of insurance coverage (91% of medical cannabis consumers reported no insurance coverage).^2^ A summary from the 2016 meeting between patient advocacy groups and the Task Force on Marijuana Legalization and Regulation reported patients can pay up to $500 a month for their medical cannabis.^3^ This financial burden is further compounded by the addition of an excise tax to cannabis that was put in place on October 17, 2018.

This type of taxation is typically applied to tobacco or alcohol, and not medications that require a prescription. Thus, the application of an excise tax to medical cannabis is unique and at odds with how Canada currently treats the taxation of medications. The taxation of medical cannabis has added a significant financial burden for patients using it to relieve chronic symptoms and creates a financial barrier, making medical cannabis inaccessible for patients who could benefit from its use. The application of these taxes can increase product costs by up to 25% for patients, depending on the province.^4^ The Arthritis Society reported that the sales and excise taxes on medical cannabis cost an average arthritis patient almost $2000/year, based on average dosage.^5^

Although some may argue that standard pharmaceutical treatments are sufficient for treatment, research does not support this. A large proportion of patients find medical cannabis to be more effective and tolerable than standard pharmaceuticals for the management of their symptoms.^6–8^ Medical cannabis also has a role as an adjunct therapy. For example, up to 52% of medical cannabis consumers were able to reduce the use of other medications with the addition of medical cannabis to their treatment regimen.^2^

Scientific evidence continues to grow and support the efficacy of cannabinoids in treating a wide range of medical symptoms and conditions. One of the conditions this extends to is for the treatment of chronic pain.^9,10^

Recently, four consensus recommendations were published in peer-reviewed journals (Sihota et al.,^11^ MacCallum et al.,^12^ Bhaskar et al.^13^ and Busse et al.^14^), outlining guidance for the administration of cannabinoid-derived therapies to treat chronic pain. The strength of these protocols lies in the fact that through four different methodological approaches, each of the research groups arrived at the recommendation that noninhaled cannabinoids can be a helpful addition to treat chronic pain in patients with insufficiently managed pain.^11–14^ All four studies highlighted a starting dose of ∼5 mg cannabidiol with guidance for titrating up slowly as well as the option to add on tetrahydrocannabinol (THC) (as low as 0.5 mg THC initially with guidance for titrating) for further pain management.^11–14^

Sihota et al.'s guidance also provides recommendations on how to taper opioids in response to a successful cannabinoid initiation.^11^ This builds off the increasingly growing number of studies that indicate cannabis is being used by patients as a substitute for conventional pharmaceuticals, such as opioids.^6–8,15–17^ In these studies, patients reported that medical cannabis improved their overall quality of life and symptoms and caused less adverse events than their previously prescribed conventional pharmaceuticals.^6–8^ By using cannabis, patients reported reductions in their daily opioid dosages and even ceased to use opioids entirely.^6,7^

In February 2022, Benedict et al. published a prospective study that found medical cannabis as an alternative treatment for chronic pain resulted in a significant reduction in chronic opioid use; after the first follow-up there was a 67.1% decrease in morphine milligram equivalents.^18^ This evidence is of increasing importance and interest, given the opioid crisis in North America. Between January and June 2021, there were 3512 apparent opioid toxicity deaths in Canada.^19^ In the April through June period alone, this equated to ∼19 deaths a day and an increase of 66% in comparison with April–June 2019.^19^ Furthermore, approximately a third of patients prescribed opioids for chronic pain misuse them and between 8% and 12% develop an opioid use disorder.^20^

Given the aforementioned evidence, cannabinoids should be incorporated as an integral part of medical treatment regimens to mitigate the number of patients who find themselves chronically using and/or misusing opioids to manage pain. Even though medical cannabis supports chronic pain management and mitigates opioid abuse, the continued taxation of medical cannabis is likely to dampen its widespread use by erecting a significant financial barrier.

It is difficult to understand the rationale to make medical cannabis unaffordable through undue taxation when it could provide patients the ability to treat their symptoms effectively without the abuse and overdose potential of opioids. We are concerned that because opioids are not taxed and are covered by government and insurance plans, patients may be choosing them even though they are less safe for long-term use. Simply put, continuing to tax medical cannabis is taxing medicine and as such, taxing people with legitimate health issues and disabilities.^21^

The lack of recognition medical cannabis faces as a proper prescription is not reflective of the amount of data that is currently available nor in line with its safety profile. According to the WHO Expert Committee on Drug Dependence, a lethal dose in a 70 kg human would be 4 g of THC and that “such a dose could not be realistically achieved in a human following oral consumption, smoking or vaporizing”.^22^ This is because cannabinoids do not directly affect respiratory or cardiovascular function,^23^ unlike opioids that significantly depress respiratory function when overdosed.^24^

At the core of this issue, it is crucial to remember it is not a choice between two equally effective and safe medications but rather for many patients, a choice between a pharmaceutical that is generously reimbursed with a significant risk of addiction and fatal overdose, and medical cannabis, an improperly taxed medicine with a large safety margin.^22^ We believe that no patient should be placed in the precarious position of having to choose between their preferred choice of medicine and putting food on the table when the financial barrier is largely the result of an unwarranted taxation of said medicine.

Therefore, it seems prudent to highlight the need for the removal of taxes (excise, GST/HST, and sales taxes) on medical cannabis to allow patients more affordable access to effective treatment options. We make this call so that medical cannabis patients may receive the same treatment that any other patient would receive with other prescriptions their health care provider has deemed necessary for their overall health.^21^ We believe now is the time for such action given the large body of scientific evidence that has shown efficacy of cannabinoids for treating chronic pain, four different protocols providing excellent guidance for initiating cannabinoid treatments and the ever-growing opioid crisis.

## Abbreviation Used

THC: tetrahydrocannabinol
